# Sex-Specific Effects of Estradiol and Progesterone in Ischemic Kidney Injury

**DOI:** 10.3390/ijms25063155

**Published:** 2024-03-09

**Authors:** Nadezda V. Andrianova, Anna A. Brezgunova, Marina I. Buyan, Ciara I. Makievskaya, Andrey I. Buyan, Kseniia S. Cherkesova, Irina B. Pevzner, Ljubava D. Zorova, Dmitry B. Zorov, Egor Y. Plotnikov, Vasily A. Popkov

**Affiliations:** 1 A.N. Belozersky Institute of Physico-Chemical Biology, Lomonosov Moscow State University, 119992 Moscow, Russiazorov@belozersky.msu.ru (D.B.Z.); plotnikov@belozersky.msu.ru (E.Y.P.); 2 Institute for Artificial Intelligence, Lomonosov Moscow State University, 119992 Moscow, Russia; 3 Faculty of Bioengineering and Bioinformatics, Lomonosov Moscow State University, 119992 Moscow, Russia; 4 Institute of Protein Research, Russian Academy of Sciences, 142290 Pushchino, Russia; 5 K.I. Skryabin Moscow State Academy of Veterinary Medicine and Biotechnology, 109472 Moscow, Russia; 6 V.I. Kulakov National Medical Research Center for Obstetrics, Gynecology and Perinatology, 117198 Moscow, Russia

**Keywords:** kidney, mitochondria, steroid hormones, hormone receptors, therapy, nephroprotection

## Abstract

The positive effects of female sex hormones, particularly estradiol and progesterone, have been observed in treatment of various pathologies. Acute kidney injury (AKI) is a common condition in hospitalized patients in which the molecular mechanisms of hormone action are poorly characterized. In this study, we investigated the influence of estradiol and progesterone on renal cells during ischemic injury. We performed both in vivo experiments on female and male rats and in vitro experiments on renal tubular cells (RTCs) obtained from the kidneys of intact animals of different sexes. Since mitochondria play an important role in the pathogenesis of AKI, we analyzed the properties of individual mitochondria in renal cells, including the area, roundness, mitochondrial membrane potential, and mitochondrial permeability transition pore (mPTP) opening time. We found that pre-treatment with progesterone or estradiol attenuated the severity of ischemia/reperfusion (I/R)-induced AKI in female rats, whereas in male rats, these hormones exacerbated renal dysfunction. We demonstrated that the mPTP opening time was higher in RTCs from female rats than that in those from male rats, which may be one of the reasons for the higher tolerance of females to ischemic injury. In RTCs from the kidneys of male rats, progesterone caused mitochondrial fragmentation, which can be associated with reduced cell viability. Thus, therapy with progesterone or estradiol displays quite different effects depending on sex, and could be only effective against ischemic AKI in females.

## 1. Introduction

The effect of various hormones, including sex hormones, on tissue resistance to damage has been known for a long time. The positive effect of female sex hormones is evidenced by the fact that many diseases occur 5–10 years later in women than in men [1,2,3]. The incidence of many groups of diseases increases after menopause, when the production of female hormones decreases significantly [4,5]. At the same time, hormone replacement therapy has a protective effect on some diseases [6]. Moreover, the protective effect of estrogens in ischemic injuries has been directly demonstrated in some organs [7,8].

As for kidney tissue, around 13.3 million people worldwide are affected by acute kidney injury (AKI) each year, resulting in 1.7 million deaths [9]. The varying etiology of AKI, the anatomy of kidney tissue, and the sensitivity of renal tubular cells to damaging factors may complicate the development of pathogenetic therapy for this disease and facilitate its progression to chronic kidney disease (CKD) [10,11]. As planned surgery is one of the most common causes of AKI, several therapeutic strategies allow the use of pre-treatment [12].

Some sex differences in susceptibility to ischemic AKI and CKD have also been identified [13,14,15]. During ischemia/reperfusion (I/R) and nephrotoxic injury, females exhibited less apoptosis, kidney injury, and fibrosis development compared with males [16]. Such nephroprotection was lost after ovariectomy and was restored via estradiol administration [17]. On the contrary, the level of renal tubular damage after I/R was lower in castrated mice compared with that in intact animals, and testosterone administration reversed this effect [18]. Progesterone and estradiol are the hormones directly associated with pregnancy and are necessary to maintain numerous functions during gestation [19]. Recently, pregnancy was shown to mitigate the severity of AKI and kidney tissue damage, possibly due to an increased tolerance of the kidney of pregnant rats to the injury [20].

This study aimed to investigate the effects of female sex hormones on AKI induced by I/R in female and male rats. We assessed the severity of AKI 24 h after renal I/R with progesterone or estradiol pre-treatment 1 h before I/R. To uncover some mechanisms of hormones’ effects on kidney cells, we evaluated the effect of progesterone and estradiol on the viability of renal tubular cells (RTCs) after ischemic injury induced by oxygen–glucose deprivation (OGD). Moreover, we assessed some characteristics of mitochondria, including mitochondrial area, shape, transmembrane potential, and the time for the induction of the mitochondrial permeability transition pore (mPTP) in RTCs from female and male rats.

## 2. Results

### 2.1. Progesterone Ameliorates the Severity of AKI in Female but Not Male Rats

**Figure 1:**
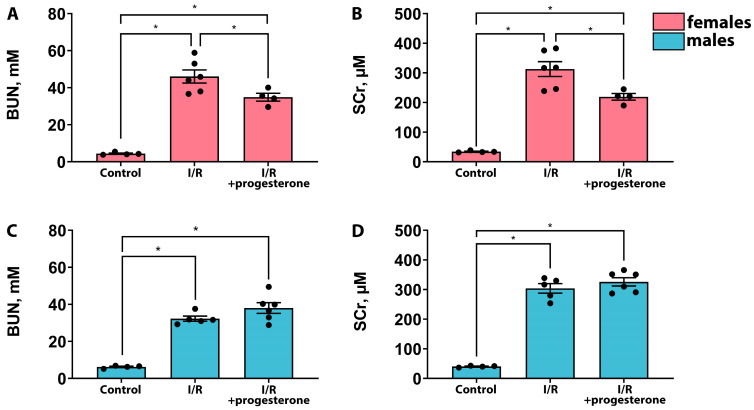
Effects of progesterone treatment on the severity of AKI in female and male rats. BUN concentration in serum of female (**A**) and male (**C**) rats 24 h after I/R. SCr concentration in serum of female (**B**) and male (**D**) rats 24 h after I/R. * *p* < 0.05 (one-way ANOVA with Tukey’s multiple comparison test).

**Figure 2:**
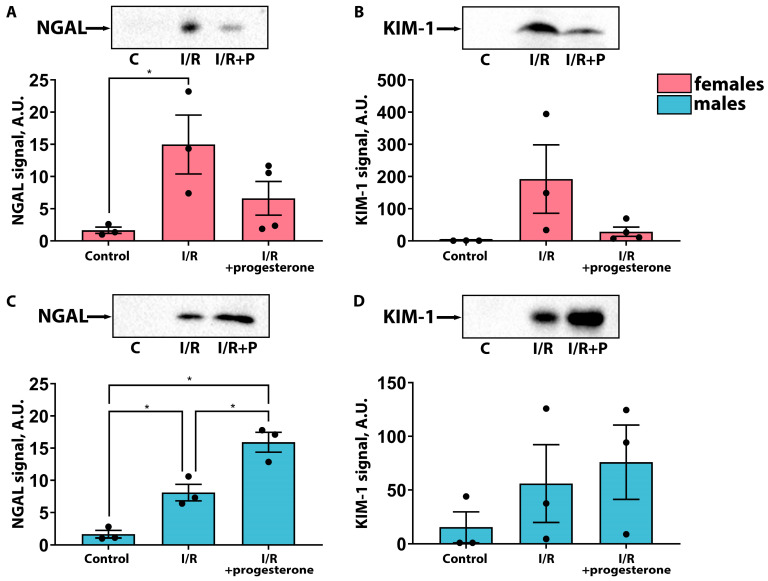
Influence of progesterone treatment on kidney injury markers in female and male rats. NGAL level in the urine of female (**A**) and male (**C**) rats 24 h after I/R measured via Western blotting. KIM-1 level in the urine of female (**B**) and male (**D**) rats 24 h after I/R measured via Western blotting. * *p* < 0.05 (one-way ANOVA with Tukey’s multiple comparison test or Kruskal–Wallis test with Dunn’s multiple comparison test).

We assessed the effects of progesterone administration 1 h before renal I/R on the severity of AKI in female and male rats. It was found that treatment with progesterone decreased blood urea nitrogen (BUN) and serum creatinine (SCr) concentrations in female rats compared with those of the “I/R” group (Figure 1A,B), indicating the amelioration of AKI. On the contrary, there was no decrease in BUN and SCr levels in male rats after progesterone administration (Figure 1C,D). These results were confirmed via an analysis of more sensitive and specific markers of renal tissue damage—neutrophil gelatinase-associated lipocalin (NGAL) and kidney injury molecule 1 (KIM-1) in urine. We revealed that these markers tended to decrease after I/R in female rats treated with progesterone (Figure 2A,B), while in male rats, progesterone administration caused an upward trend for these markers (Figure 2C,D). Thus, we demonstrated that progesterone had a protective effect against AKI in female rats, while in male rats it had no effect or even worsened kidney damage during I/R.

### 2.2. Estradiol Mitigates the Severity of AKI in Female Rats but Worsens It in Male Rats

**Figure 3:**
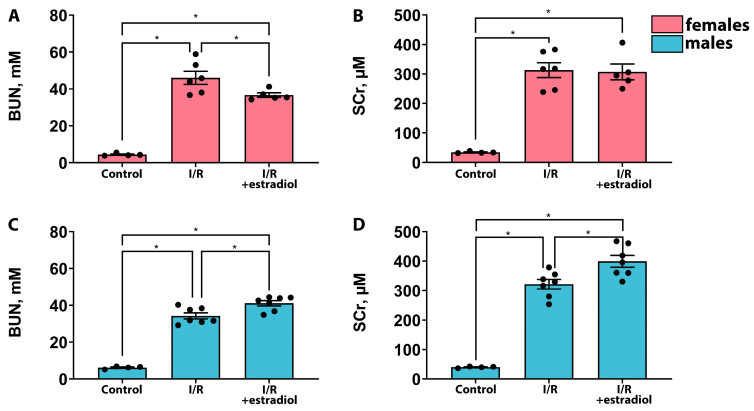
Effects of estradiol therapy on the severity of AKI in the female and male rats. BUN concentration in serum of female (**A**) and male (**C**) rats 24 h after I/R. SCr concentration in serum of female (**B**) and male (**D**) rats 24 h after I/R. * *p* < 0.05 (one-way ANOVA with Tukey’s multiple comparison test).

**Figure 4:**
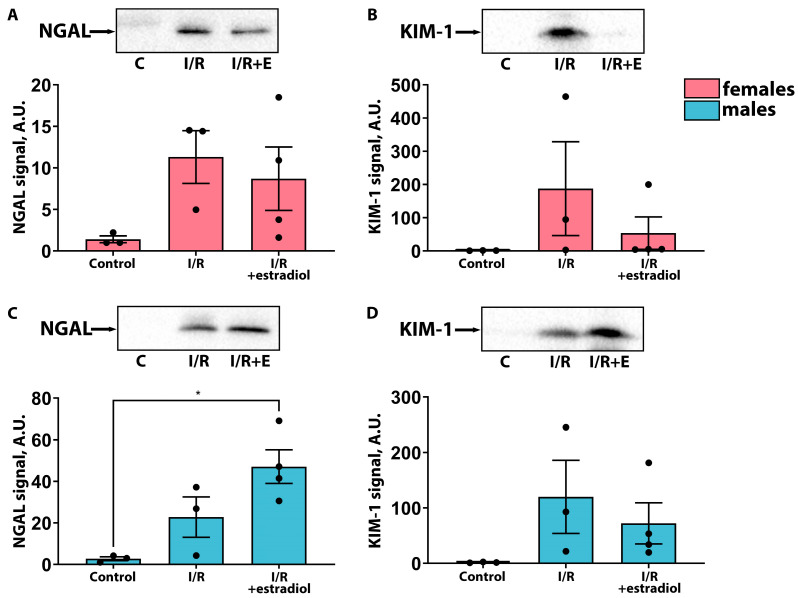
Influence of estradiol therapy on kidney injury markers in female and male rats. NGAL level in the urine of female (**A**) and male (**C**) rats 24 h after I/R measured via Western blotting. KIM-1 level in the urine of female (**B**) and male (**D**) rats 24 h after I/R measured va Western blotting. * *p* < 0.05 (one-way ANOVA with Tukey’s multiple comparison test or Kruskal–Wallis test with Dunn’s multiple comparisons).

The effects of estradiol on the severity of AKI were also assessed in rats of different sexes. Similar to the effects of progesterone, estradiol reduced the severity of AKI in female rats, resulting in a decrease in the BUN concentration (Figure 3A). However, the SCr level in female rats remained unaltered by estradiol therapy (Figure 3B). In male rats, pre-treatment with 5 mg/kg of estradiol resulted in a significant increase in BUN and SCr levels compared with those of the “I/R” group (Figure 3C,D). An analysis of urinary markers of renal damage NGAL and KIM-1 confirmed that estradiol treatment ameliorated AKI severity in female rats and aggravated it in male rats (Figure 4). Thus, the administration of estradiol before injury mitigated the effects of AKI in female rats, whereas in male rats, such therapy worsened kidney function.

### 2.3. Progesterone Causes Mitochondrial Fragmentation in Kidney Cells

**Figure 5:**
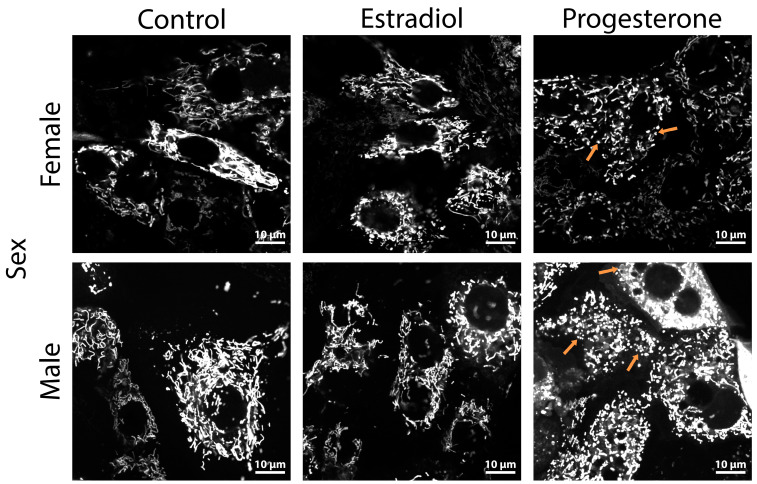
Mitochondrial structure in RTCs from female and male rats incubated with estradiol or progesterone. Representative confocal images of primary RTC cultures loaded with TMRE. Orange arrows point out mitochondrial fragmentation. Scale bar: 10 μm.

**Figure 6:**
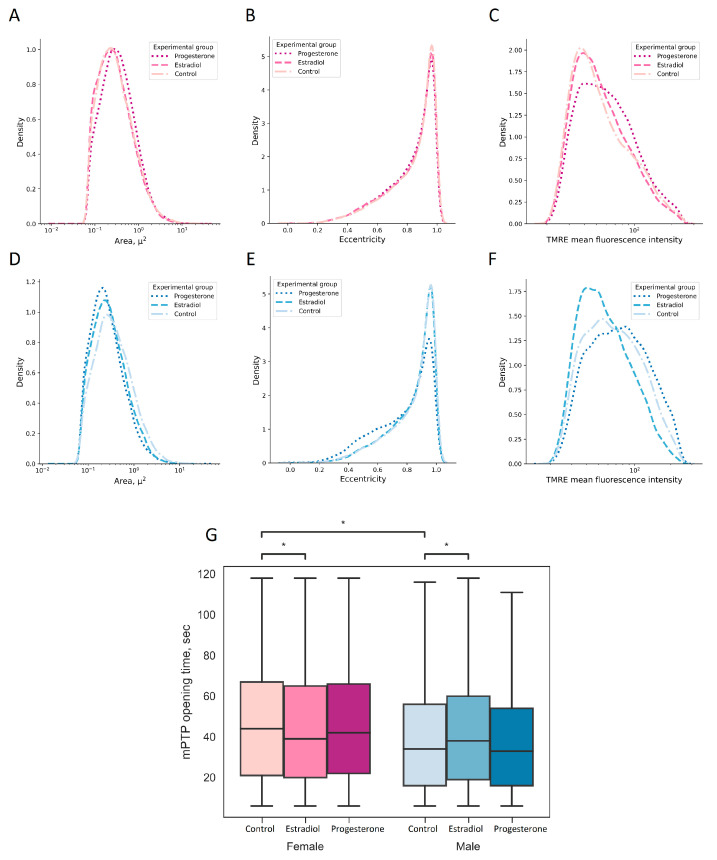
Effects of estradiol or progesterone treatment on structural and functional characteristics of individual mitochondria in RTCs obtained from female and male rats. Distribution of mitochondrial area in RTCs from female (**A**) and male (**D**) rats; distribution of eccentricity of mitochondria of RTCs from female (**B**) and male (**E**) rats; distribution of TMRE mean fluorescence intensity of mitochondria of RTCs from female (**C**) and male (**F**) rats; (**G**) mPTP opening time. * *p* < 0.05 (Mann–Whitney test with Bonferroni correction for multiple comparisons).

Mitochondria play a crucial role in AKI pathogenesis, as these organelles control energy metabolism and regulate cell death [21]. In this regard, we examined the key characteristics of mitochondria in primary cultures of RTCs obtained from the kidneys of female and male rats. It was found that incubation with progesterone resulted in mitochondrial fragmentation in RTCs derived from male rats (Figure 5). Cultures of RTCs from female rats also exhibited mitochondrial fission after incubation with progesterone, but to a lesser extent than those from male rats did (Figure 5). The distributions of mitochondrial area, eccentricity, and TMRE fluorescence intensity in RTCs from female rats in all experimental groups were asymmetric in a similar way; however, it was observed that incubation with progesterone resulted in a tendency of mitochondria to increase with higher membrane potential (Figure 6A–C). Male RTCs demonstrated an increase in the number of small round-shape mitochondria after progesterone treatment (Figure 6D,E), indicating mitochondrial fragmentation. Additionally, in RTC cultures from male rats, we observed mitochondria with a higher TMRE fluorescence intensity after incubation with progesterone, while incubation with estradiol resulted in a decrease in the mitochondrial membrane potential (Figure 6F).

In addition, the mPTP opening time, which is an important feature of mitochondria reflecting their tolerance to damaging factors, was analyzed. The opening of the mPTP was induced via the generation of ROS caused by the long-term excitation of TMRE by a green laser. Mitochondrial permeability transition results in a rapid drop in mitochondrial membrane potential, causing TMRE release from the mitochondria. When analyzing such a decrease in TMRE fluorescence, we observed that the mPTP opening time was longer in RTCs from female rats compared with that in those of male rats (Figure 6G), which may indicate higher tolerance to the mPTP induction of cells from female rats. Incubation with progesterone did not affect the mPTP opening time in cells both from female and male rats, while incubation with estradiol accelerated mPTP opening in females, and delayed it in males.

### 2.4. Effects of Progesterone and Estradiol on the Viability of RTCs from Female and Male Rats

**Figure 7:**
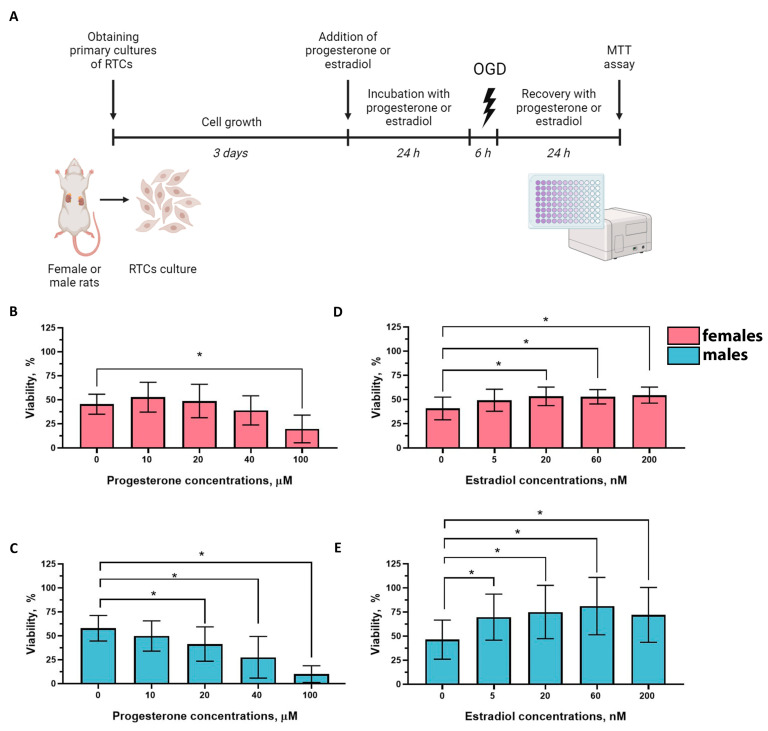
Effects of progesterone and estradiol on the viability of the RTCs, obtained from female (red charts) and male (blue charts) rats, after OGD in vitro. (**A**) Experimental design; (**B**) viability of the female RTCs incubated with progesterone and exposed to OGD; (**C**) viability of the female RTCs incubated with estradiol and exposed to OGD; (**D**) effects of progesterone on the viability of male RTCs exposed to OGD; (**E**) effects of estradiol on the viability of male RTCs exposed to OGD. * *p* < 0.05 (one-way ANOVA with Tukey’s multiple comparison test).

To assess the effects of progesterone and estradiol on ischemic tolerance in vitro (Figure 7), we analyzed the OGD-induced cell death of RTCs obtained from the kidneys of female and male rats (Figure 7A). We revealed that progesterone did not significantly change the viability of RTCs from female rats in the therapeutic concentration range (Figure 7B). However, the same concentrations of progesterone had a deterioration effect on the RTCs from male rats (Figure 7C). Estradiol, in contrast, showed a positive effect on the viability of the OGD-exposed RTCs from both female (Figure 7D) and male kidneys (Figure 7E).

## 3. Discussion

The potential of hormone therapy to alleviate various pathological conditions has long received considerable scientific and clinical interest, including therapeutic options for sex hormones. It is known that males are more sensitive to ischemic injuries [22,23]. For the kidney, I/R was shown to cause more significant damage to male rats compared with females [24], while female rats lost their protection after ovariectomy [17]. It is considered that sex hormones play a pivotal role in sex-dependent susceptibility to AKI and its progression to CKD [25].

In this study, we investigated the effects of the female sex hormones, progesterone and estradiol, on kidneys in female and male rats under normal conditions and after I/R. We showed that these hormones have nephroprotective effects in the case of female rats and predominantly negative effects in male rats (Figure 1, Figure 2, Figure 3 and Figure 4). Previously, it was demonstrated that a single administration of exogenous estradiol resulted in a significant reduction in pyroptosis-associated inflammation in the damaged kidney of non-ovariectomized females [26] and reduced the severity of renal injury in ovariectomized females [27,28,29]. Regarding progesterone, it has been shown that the administration of this hormone to ovariectomized female rats protected against glomerulosclerosis, decreased profibrotic factors, and eliminated other manifestations of nephropathy in a model of diabetes-associated kidney damage [30].

Several molecular mechanisms underlying effects of progesterone and estradiol in various diseases have been suggested, including kidney pathologies. Specifically, one of the mechanisms contributing to the positive effects of exogenous estradiol on the severity of AKI in female rats can be realized through the inactivation of the transforming growth factor β (TGF-β) I receptor (TGF-βRI)-SMAD signaling pathway [17]. The inhibition of this pathway not only decreases inflammation following renal ischemia but also leads to a notable decrease in the levels of TGF-β, one of the key regulators of kidney fibrosis development after injury [17]. There is another way to achieve beneficial effects, namely by influencing endothelial dysfunction, which is one of the most important mechanisms of AKI. The administration of exogenous estradiol activates the phosphoinositide 3-kinase (PI3K)/Akt/endothelial NO synthase (eNOS) pathway, thereby mitigating the severity of AKI through the normalization of NO synthesis [31].

The influence of female sex hormones on males has only been described in very few studies. The administration of exogenous estradiol to male rats did not have the same nephroprotective effect against injury induced by renal I/R as it did in female rats [32]. As to neural cells, the incubation of cortical astrocytes obtained from male rats with progesterone or estradiol resulted in decreased viability and increased apoptosis levels, while cortical astrocytes from female rats, on the contrary, exhibited an increase in viability after incubation with hormones [33]. In our study, we revealed the same difference in the effects of progesterone and estradiol on the severity of renal damage in male and female rats. We showed that in male rats, progesterone and estradiol do not protect against I/R-induced AKI, but further worsen the condition in male rats, while the opposite effect was females.

**Figure 8:**
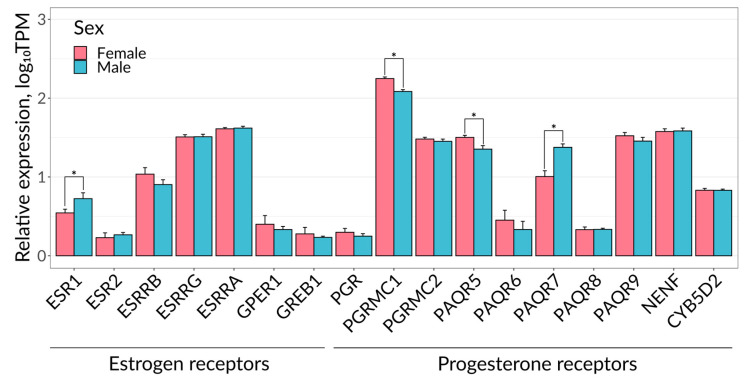
Expression profiles of estrogen and progesterone receptor genes in kidney cells. Error bars indicate the standard deviation of the four biological replicates. * *p* < 0.05 (two-sided Wilcoxon rank sum test). TPM—transcripts per million.

One of the possible explanations for the sex-dependent differences could be the variability in the expression of progesterone and estradiol receptors in female and male kidneys. Indeed, our analysis of the expression of 17 genes of the progesterone and estrogen receptor in available RNA-seq data [34] revealed that progesterone receptor membrane component 1 (PGRMC1) and progestin and adipoQ receptor 5 (PAQR5) were significantly upregulated in female compared with male kidneys, whereas ESR1 and progestin and adipoQ receptor 7 (PAQR7) expression was higher in males (Figure 8). Progesterone and estradiol receptors are important for the functioning of renal tissue under both normal and pathological conditions [35] and perform their function as transcription factors for numerous genes [36]. In particular, the knockout of the estradiol receptor ESR1 in epithelial cells led to the dysregulation of TGF-β expression and the activation of the inflammatory response, while the overexpression of ESR1 reduced the level of ischemic damage through the inhibition of the TGF-βRI-SMAD cascade [17]. Earlier, we proposed that the lack of progesterone effects in unilateral ureteral obstruction may be due to the decreased expression of PAQR5 in the kidney [37].

Mitochondrial functions are also affected by sex hormones, while mitochondria play one of the key roles in the pathogenesis of ischemic damage [38]. The sex-dependent response of mitochondria and related cellular systems to ischemia and subsequent reperfusion may also be the cause of the different susceptibilities between males and females to injury. It was shown that mitochondria from females and males exhibit some differences in functional features [39]. Particularly, the mitochondria of females show more efficient respiration and adenosine triphosphate (ATP) production [40], as well as more effective antioxidant systems, providing greater protection against oxidative stress [41].

Estradiol and progesterone may regulate mitochondrial function indirectly through the activation of the expression of master regulators of respiratory activity (e.g., nuclear respiratory factors, NRFs) and mitochondrial biogenesis (e.g., peroxisome proliferator-activated receptor gamma coactivator 1-α, PGC1α) [42,43]. The expression of these master regulators directly affected the mitochondria, the functioning, and efficiency of the electron transfer chain, the level of oxidative stress, and the induction of cytochrome c-induced apoptosis [44]. Mitochondria isolated from rats treated with progesterone or estradiol displayed enhanced metabolic rates and increased respiratory function. These alterations were associated with an increased expression of electron transfer chain complex IV, a decreased rate of reactive oxygen production, and reduced lipid peroxidation [45].

Apart from the bioenergetic functions and regulation of apoptosis, mitochondrial dynamics is an important feature of mitochondria that affects the development of pathologies [46]. An imbalance between the fission and fusion of mitochondria can lead to a variety of diseases [47]. During AKI, a redundant fission of mitochondria occurs, accompanied by the expression of mitochondrial fission 1 protein (FIS1) and Drp1 [48]. The inhibition of mitochondrial fragmentation prevents the progression of AKI to CKD [49]. That is why we assessed mitochondrial dynamics and analyzed the effects of sex and hormone treatment to them. We found that incubation with progesterone causes mitochondrial fragmentation in males but not females (Figure 5 and Figure 6).

Moreover, we assessed the sex differences in estradiol and progesterone effects on an important indicator of mitochondrial functioning, the induction of mPTP (Figure 6E,F). mPTP is a nonspecific, highly conductive channel that increases the permeability of the inner mitochondrial membrane to ions and small solutes [50,51]. Elevated ROS levels are one of the most important triggers for mPTP opening [52,53]. The irreversible damage to several organs that occurs during oxidative stress induced by ischemia and reperfusion is at least partially mediated by the opening of mPTP [54,55,56], which leads to a drop in transmembrane mitochondrial potential and subsequent cell death. We showed that female-derived RTCs have a higher pore opening time than do male-derived RTCs (Figure 6G), indicating higher tolerance to damage in female RTCs. These effects may be partially attributed to the greater capacity for calcium retention in female mitochondria than that in male mitochondria [57]. While incubation with progesterone and estradiol had a minimal effect on the mPTP opening time in female RTCs, progesterone exhibited a rather negative effect in male RTCs (Figure 6G).

## 4. Materials and Methods

### 4.1. Animals

Experiments were performed on female (*n* = 35) and male (*n* = 35) young outbred Wistar rats (3–4-month-old, 300–400 g). Animal protocols were evaluated and approved by the animal ethics committee of A.N. Belozersky Institute of Physico-Chemical Biology Lomonosov Moscow State University, Protocol 3/19 from 18 March 2019. All procedures were in accordance with the “Animal Research: Reporting of In Vivo Experiments” (ARRIVE) guidelines. The animals had unlimited access to food and water and were kept in cages in a temperature-controlled environment (20 ± 1 °C) under a 12/12 h light/dark regime. All female rats used in our experiments were in estrus on the day of I/R, as determined via vaginal smear microscopy.

### 4.2. Renal I/R Protocol

Some rats, 1 h before I/R, received an intraperitoneal injection of 10 mg/kg of progesterone (Sigma-Aldrich, St. Louis, MO, USA) or 5 mg/kg of estradiol (Sigma-Aldrich, USA) dissolved in 70% propylene glycol (Sigma-Aldrich, USA) solution. The doses of hormones were chosen based on previous studies [30,58,59]. The number of animals in the control group was *n* = 4, in the “I/R” group, this was *n* = 6, and in each I/R group with progesterone or estradiol treatment, this was *n* = 6. To perform renal I/R, rats were anesthetized and subjected to 40 min of warm ischemia of the left kidney, as previously described [60]. In brief, the left renal vascular bundle was occluded with a microvascular clip for 40 min. Nephrectomy of the right kidney was performed simultaneously with ischemia of the left one. After 40 min of ischemia, circulation was restored by removing the microvascular clip. During the surgery procedure, the body temperature of the rats was maintained at 37 ± 0.5 °C. To confirm AKI, urine and blood samples were taken 24 h after I/R.

### 4.3. Serum Analysis

To determine BUN and SCr concentrations, samples of the blood were taken from the carotid artery. After 15 min at room temperature, the clot was removed via centrifugation at 2000× *g* for 5 min at 2–4 °C. The resulting serum was analyzed for SCr and BUN using AU480 Chemistry System (Beckman Coulter, Brea, CA, USA).

### 4.4. Western Blotting

To analyze urinary NGAL and KIM-1 levels, urine samples were centrifuged at 10,000× *g* for 5 min, mixed with 2× sample buffer containing 10% 2-mercaptoethanol, and boiled for 5 min. Then, samples were centrifuged at 10,000× *g* for 5 min and loaded onto 15% Tris-glycine polyacrylamide gel in amounts of 20 μL to each lane. After electrophoresis, proteins were transferred onto PVDF membranes (Amersham Pharmacia Biotech, Buckinghamshire, UK). Membranes were blocked with 5% non-fat milk in PBS with 0.05% Tween-20 and subsequently incubated with primary rabbit antibodies against NGAL (ab63929, Abcam, Cambridge, UK) or primary mouse antibodies against KIM-1 (MAA785Ra21, Cloud Clone Corp., Wuhan, China). Membranes were then incubated with secondary anti-rabbit or anti-mouse IgG antibodies conjugated with horseradish peroxidase (Jackson ImmunoResearch, West Grove, PA, USA) and probed with an Advansta Western Bright ECL kit (Advansta, San Jose, CA, USA). Detection was performed using V3 Western Blot Imager (BioRad, Hercules, CA, USA).

### 4.5. Primary RTCs Cultures

For in vitro experiments, primary cultures of RTCs were isolated from the kidneys of female (*n* = 3) and male (*n* = 3) rats, as previously described [61]. The rats were euthanized, and kidneys were aseptically isolated, cut into small pieces, and incubated with 0.25% collagenase II type (Gibco, Billings, MT, USA, ThermoFisher Scientific, Waltham, MA, USA) in DMEM/F12 medium without sodium bicarbonate (GE healthcare, Waukesha, WI, USA) at 37 °C for 30 min. Kidney pieces were pipetted, and the resulting suspension was centrifuged for 5 min at 400× *g* to pellet the tubular fraction. The pellet was resuspended in a DMEM/F12 complete culture medium (PanEco, Moscow, Russia) with 10% fetal bovine serum (FBS) (FB-1001, BioSera, Cholet, France). The resulting renal tubules were plated on cultural dishes; the medium was changed 48 h after seeding to remove cellular debris. To study the effects of hormones, progesterone (Sigma-Aldrich, USA) or estradiol (Sigma-Aldrich, USA) was added to cell cultures for 24 h when they reached 70% of the monolayer on the 3rd day of cultivation. Each experiment performed on RTC cultures was repeated three times.

### 4.6. Analysis of Characteristics of Individual Mitochondria

**Figure 9:**
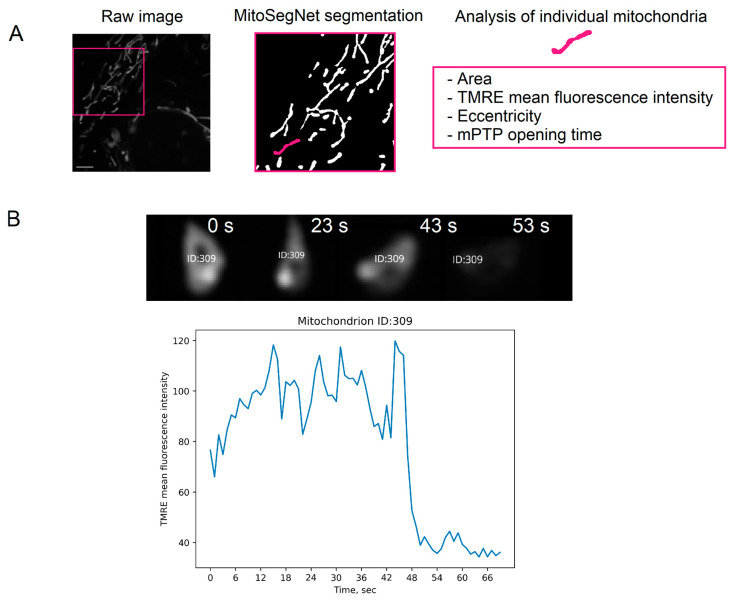
The scheme of analysis of the morphological and functional characteristics of individual mitochondria in RTCs isolated from the kidneys of female and male rats. (**A**) Confocal microscope’s video processing pipeline including mitochondrial segmentation with MitoSegNet, tracking with STracking, and the analysis of characteristics such as area, TMRE mean fluorescence intensity, eccentricity, and the mPTP opening time. Scale bar: 10 μm; (**B**) An example of the mPTP opening. A sharp drop in the TMRE mean fluorescence intensity in the mitochondria between 40 and 48 s in this example was detected as the mPTP opening, which was also visually noticeable in the video of these mitochondria.

RTCs isolated from the kidneys of female and male rats were incubated with 40 μM progesterone or 100 nM estradiol for 24 h. Then, cells were washed and stained with TMRE (ThermoFisher Scientific, Waltham, MA, USA, Invitrogen, Carlsbad, CA, USA), a fluorescent, positively charged dye that accumulates in normally functioning mitochondria due to their internal negative charge [62]. For staining, a 200 nM TMRE solution was prepared in DMEM/F12 medium without sodium bicarbonate (GE healthcare, USA). Cells were incubated with dye solution for 30 min at 37 °C and then washed with DMEM/F12 without sodium bicarbonate. To analyze individual mitochondria, time lapse videos (120 s per video, 1 frame per 1 s, LSM Scan Speed—7, Airyscan super resolution mode) were acquired using an LSM900 confocal microscope (Carl Zeiss, Baden-Württemberg, Germany). Time-lapse videos were converted and binary mitochondrial segmentation masks were obtained frame by frame using the MitoSegNet neural network model (accessed on 31 January 2024) [63]. Using the first frame of the obtained time lapse videos, the individual characteristics of the mitochondria were analyzed: area, mean TMRE fluorescence intensity, and eccentricity, which characterizes the degree of roundness of mitochondria (Figure 9A). To analyze the mPTP opening time, both the raw frames and the predicted mitochondrial segmentation masks were further processed using STracking (accessed on 31 January 2024) [64], an open-source library implemented in Python version 3.9 that allows the tracking of cellular compartments. The obtained data on the movement of mitochondria over time and changes in the brightness of their pixels were used for further analysis of the dynamics of mPTP opening (Figure 9B).

### 4.7. Cell Viability

RTC cultures obtained from the kidneys of female or male rats were cultured in 96-well plates for 3 days and then incubated with 10–100 μM progesterone (Sigma-Aldrich, USA) or 5–200 nM estradiol (Sigma-Aldrich, USA) for 24 h, based on previous studies [17,65,66,67,68,69]. After pre-treatment with hormones, cells were subjected to OGD, during which DMEM/F12 complete culture medium (PanEco, Moscow, Russia) with 10% FBS (FB-1001, BioSera, France) was replaced by sterile Dulbecco’s phosphate-buffered saline (DPBS) without glucose (Corning Inc., Corning, NY, USA). RTCs were put into a Galaxy 170R multi-gas incubator (Eppendorf/NewBrunswick, Hamburg, Germany) with 1% O_2_ and 0% CO_2_ for 6 h. Then, the normoxic conditions and DMEM/F12 complete culture medium with 10% FBS with or without progesterone or estradiol were returned to. After 24 h, an MTT assay (PanEco, Russia) was carried out to analyze the viability of cells. Absorption was measured at 540 nm using a Zenyth 3100 plate multimode detector (Anthos Labtec, Wals, Austria). Wells with cells incubated with H_2_O for 24 h were used as a negative control.

### 4.8. Bioinformatics Analysis

TPM expression values for genes related to estrogen and progesterone receptor activity were calculated from gene-level read counts downloaded from GEO GSE175854 [41]. Exonic gene lengths used for calculating TPM were obtained via gencode M25 comprehensive gene annotation [70], as well as using the makeTxDbFromGFF and exonsBy functions of the GenomicFeatures R package version 1.38.2 [71]. Differences in estrogen and progesterone receptor gene expression were tested using a two-sided Wilcoxon rank sum test.

### 4.9. Statistical Analysis

Statistical data analysis was performed using GraphPad Prism 9 software (GraphPad Software Inc., Boston, MA, USA). The data were tested for normal distribution using the Shapiro–Wilk test. Parametric data were analyzed via a one-way ANOVA with Tukey’s multiple comparisons test, while non-parametric data were analyzed using the Kruskal–Wallis test with Dunn’s multiple comparisons or the Mann–Whitney test with Bonferroni’ correction for multiple comparisons. Values are presented as mean ± SEM. *p* < 0.05 was considered statistically significant.

## 5. Conclusions

We showed that the effect of the female sex hormones progesterone and estradiol on the ischemic tolerance of kidney cells is largely dependent on the animal’s sex. Single administration of these hormones showed the greatest nephroprotective efficacy in female kidneys, whereas in male cells they demonstrated no beneficial effects or even worsened renal function after ischemia. We relate such a difference in action to the different representations of a set of receptors for these hormones in males and females, whereby the pattern of signaling they elicit may differ markedly both as a function of sex and in different organs. These data should be taken into account when developing hormone therapy for AKI and when predicting the risk of AKI in patients of different sexes or with different hormonal statuses. We hope that our findings can contribute to the development of an effective therapy for AKI. In future studies, a longer duration of hormone therapy or combined therapy could be used, and the possibility of using male sex hormones should be investigated.

## Data Availability

The data that support the findings of this study are available from the corresponding author upon reasonable request.
